# Frit flies of Turkey with descriptions of two new species and new records (Diptera, Chloropidae)

**DOI:** 10.3897/zookeys.667.10758

**Published:** 2017-04-10

**Authors:** Štěpán Kubík, Miroslav Barták

**Affiliations:** 1 Czech University of Life Sciences, Faculty of Agrobiology, Food and Natural Resources, Department of Zoology and Fisheries, 165 00 Praha - Suchdol, Czech Republic

**Keywords:** Acalyptratae, *Dicraeus*, Diptera, *Meromyza*, Turkey

## Abstract

Faunistic records for 88 frit flies species from southwestern Turkey (Muğla province) and from Samsun (north Turkey) are given. Two species, *Dicraeus civeleki* **sp. n.**, and *Meromyza samsunensis* **sp. n.**, are described as new to science. Altogether, nine genera (*Calamoncosis*, *Eribolus*, *Gaurax*, *Incertella*, *Speccafrons*, *Trachysiphonella*, *Chloropsina*, *Eutropha*, and *Lagaroceras*) and 46 species are recorded for the first time from Turkey.

## Introduction

Frit flies (Diptera, Chloropidae) are small to medium sized flies, adult body length 1.5–5.0 mm, rarely larger, with reduced bristling. Body colour very variable, most species are entirely black, and often with metallic sheen (subfamily Oscinellinae, Siphonellopsinae, Rhodesiellinae), whereas some species are yellow with black, red or brown longitudinal stripes on the scutum (subfamily Chloropinae). The adults occur in various marshy habitats, in deciduous woods, in damp meadows and in open areas. Chloropid larvae have varied feeding habits. Many species are phytophagous, and some of those damage cereals and other grasses. There are also saprophagous species, a few species that have been bred from fungi, and some predaceous species.

The family Chloropidae has not been an object of focused investigation in Turkey. Only two species, *Scoliophthalmus civeleki* and *Elachiptera bimaculata*, were included in the first Turkish checklist of Diptera (Koçak and Kemal 2009). Nartshuk (2012) summarized all published historical data, identified several specimens from Turkey, and published a more complete list in which she listed 64 species from 31 genera and 4 subfamilies. Koçak and Kemal (2013) took over the list of species from Nartshuk (2012) but forgot to include the work of Deeming and Al-Dhafer (2012) with the first record of *Rhodesiella fedtshenkoi* from Turkey. Kubik et al (2016) described *Tricimba dursuni* from Turkey as new to science. Two other species described as new to science in the current paper and 46 species recorded for the first time from Turkey increasing the total number of known Turkish species to 114.

## Materials and methods

The studied material, unless stated otherwise, was collected between 2011–2015 by M. Barták and Š. Kubík, and it is deposited in the collection of the Czech University of Life Sciences, Prague. It originates from southwestern and northern Turkey, mainly from the Muğla province and, to a lesser extent, also from the city of Samsun (Samsun province). The specimens were collected by Malaise traps (MT) and yellow and white pan water traps (PT), or they were swept from vegetation (SW). Most of the specimens were originally preserved in alcohol and were dried and mounted later on using the method described by Barták (1997). The genitalia of the described species here were macerated in 10 % KOH (24 hours, room temperature) and later stored together with the specimens on plastic tags and fixed with butyl-methacrylate copolymer of methyl-methacrylate, xylene. The genitalia and individual species were photographed using a Nikon D300 digital camera mounted on a Nikon SMZ-U microscope and images were edited with the computer software NIS-Elements 3.0. On average, each final image is a stack from 15 layers. Images were improved using the software Adobe Photoshop, genitalia served as models for outline of hand drawn illustrations; details were added by direct observation of the genitalia.

The morphological terms used here follow Merz and Haenni (2000). The distribution of species, unless stated otherwise, was taken from Nartshuk (2012, 2013). The species recorded here with for the first time from Turkey are marked by an asterisk and males, females are abbreviated M, F, respectively.

## List of species

### Subfamily: Siphonellopsinae

#### Apotropina longepilosa

Taxon classificationAnimaliaDipteraChloropidae

(Strobl, 1893)

##### Material examined.

Samsun, University campus, 22.vi–4.vii.2014, 3M.

##### Distribution.

Widely distributed in the southern Palaearctic Region, from Europe to the Russian Far East and Mongolia.

#### Siphonellopsis lacteibasis

Taxon classificationAnimaliaDipteraChloropidae

Strobl, 1906

##### Material examined.

Turkey: Akyaka, river bank + salty meadow, 37°03'16''N, 28°19'57"E, 16.–27.v.2011, 2M and 1F.

##### Distribution.

From southern Europe and North Africa to Central Asia.

### Subfamily: Rhodesiellinae

#### Rhodesiella fedtshenkoi

Taxon classificationAnimaliaDipteraChloropidae

Nartshuk, 1978

##### Material examined.

Turkey: 8 km S of Çine, river bank, 68 m, 37°32'34"N, 28°03'46"E, 21.ix.2012, 6M and 5F; Turkey: Toparlar, lowland forest, 36°58'39"N, 28°39'30"E, sweeping, 5.–7.5.2013, 4M and 2F.

##### Distribution.

the species was described from Kyrgyzstan and further recorded from Japan, Yemen, Saudi Arabia, Tunisia, Greece, Macedonia and Cyprus. Deeming and Al-Dhafer (2012) recorded this species from Turkey for the first time.

#### Scoliophthalmus civeleki

Taxon classificationAnimaliaDipteraChloropidae

Deeming, 2006

##### Material examined.

Turkey: Akyaka, pasture, 4 m, 37°03'09"N, 28°20'17"E, 23.–27.ix.2012, 2M.

##### Distribution.

originally described and hitherto known only from Turkey.

#### * Scoliophthalmus trapezoides

Taxon classificationAnimaliaDipteraChloropidae

Becker, 1903

##### Material examined.

Turkey: Akyaka, pasture, 4 m, 37°03'09"N, 28°20'17"E, 23.–27.ix.2012, 1M.

##### Distribution.

described from Egypt and further recorded from Kenya, Uganda, Tanzania, Zambia, Mozambique, Senegal, Burkina Faso, Nigeria, Cameroun, South Africa, Yemen, Saudi Arabia, Israel and Cyprus.

### Subfamily: Oscinellinae

#### Aphanotrigonum bicolor

Taxon classificationAnimaliaDipteraChloropidae

Nartshuk, 1964

##### Material examined.

Turkey: Akyaka, forest, 37°03'16"N, 28°19'35"E, 30.4.–9.5.2013, 30 m, 2M; Turkey: Akyaka, 40 m, forest, SW + PT, 37°03'21"N, 28°19'09"E, 16.–27.v.2011, 1M; Samsun, University campus, 22.vi–4.vii.2014, 4M.

##### Distribution.

southern Palaearctc Region, from Hungary to Central Asia.

#### Aphanotrigonum femorellum

Taxon classificationAnimaliaDipteraChloropidae

(Collin, 1946)

##### Material examined.

Turkey: Akyaka, pasture, 37°03'19"N, 28°20'07"E, 28.4.–8.5.2013, 6 m, 5M and 2F; Turkey: Akyaka, pasture, 4 m, 37°03'09"N, 28°20'17"E, 23.–27.ix.2012, 14M and 18F; Samsun, University campus, 22.vi–4.vii.2014, 1M.

##### Distribution.

a widely distributed but rare Palaearctic species, known from Europe and North Africa to Oman and Mongolia.

#### * Aphanotrigonum inerme

Taxon classificationAnimaliaDipteraChloropidae

Collin, 1946

##### Material examined.

Turkey: Akyaka, salty meadow, SW + PT, 37°02'53"N, 28°19'39"E, 28.4.–9.5.2013, 3M; Turkey: Toparlar, lowland forest, 36°58'39"N, 28°39'30"E, sweeping, 5.–7.5.2013, 2M.

##### Distribution.

West Palaearctic species.

#### * Aphanotrigonum parahastatum

Taxon classificationAnimaliaDipteraChloropidae

Dely-Draskovits, 1981

##### Material examined.

Turkey: Gökçeova Gölü, lake shore, 1 750 m, 37°03'42.52"N, 28°48'28.42"E, 20.ix.2012, 12M and 14F; Turkey: 8 km S of Çine, river bank, 68 m, 37°32'34"N, 28°03'46"E, 21.ix.2012, 18M and 7F; Turkey: Muğla, University, campus, PT, 700 m, 37°09'42"N, 28°22'21"E, 21.–24.ix.2012, 10M and 14F,: Samsun, University campus, 22.vi–4.vii.2014, 1M.

##### Distribution.

a mediterranean species, known from the North Africa, Greek mainland, French mainland, Crete and Bulgaria.

#### * Calamoncosis duinensis

Taxon classificationAnimaliaDipteraChloropidae

(Strobl, 1909)

##### Material examined.

Turkey: Akyaka, river bank + salty meadow, 37°03'16"N, 28°19'57"E, 16.–27.v.2011, 2M and 1F; Turkey: Akyaka, pasture, 4 m, 37°03'09"N, 28°20'17"E, 23.–27.ix.2012, 3M; Turkey: Akyaka, salty meadow, SW + PT, 37°02'53"N, 28°19'39"E, 28.4.–9.5.2013, 2M and 3F; Turkey: Toparlar, lowland forest, 36°58'39"N, 28°39'30"E, sweeping, 5.–7.5.2013, 4M.

##### Distribution.

a widely distributed Palaearctic species.

#### Conioscinella frontella

Taxon classificationAnimaliaDipteraChloropidae

(Fallén, 1820)

##### Material examined.

Turkey: Muğla, University campus, MT, 720 m, 37°09'42"N, 28°22'13"E, H. Kavak, 26.v.–26.vi.2015, 2M.

##### Distribution.

a widely distributed Palaearctic species, known from Europe to Israel and Mongolia.

#### * Dicraeus (Dicraeus)

Taxon classificationAnimaliaDipteraChloropidae

agropyri Nartshuk, 1964

##### Material examined.

Turkey: 13km NE of Muğla, pasture/pine wood, 1200 m, 37°14'50"N, 28°30'E, 23.–27.vi.2015, 2M.

##### Distribution.

the species is known from Russia East, Russia South, Ukraine and East Palaearctic.

#### * Dicraeus beschovskii

Taxon classificationAnimaliaDipteraChloropidae

Nartshuk, 2010

##### Material examined.

Turkey: Akyaka, pasture, 37°03'19"N, 28°20'07"E, 28.4.–8.5.2013, 6 m, 2M; Turkey: Toparlar, lowland forest, 36°58'39"N, 28°39'30"E, sweeping, 5.–7.5.2013, 2M and 1F, Turkey: Muğla, University campus, MT, 720 m, 37°09'42"N, 28°22'13"E, H. Kavak, 26.v.–26.vi.2015, 2M.

##### Distribution.

described and hitherto known only from Greece.

#### Dicraeus raptus

Taxon classificationAnimaliaDipteraChloropidae

(Holiday, 1838)

##### Material examined.

Turkey: 12km SW of Muğla, *Ferula communis*, 660 m, 37°07'40"N, 28°16'28"E, 23.v. 2011, 1M; Turkey: Muğla, University campus, MT, 720 m, 37°09'42"N, 28°22'13"E, H. Kavak, 26.v.–26.vi.2015, 2M.

##### Distribution.

this species was recorded from West Europe and from the Crimea.

#### Dicraeus tibialis

Taxon classificationAnimaliaDipteraChloropidae

(Macquart, 1835)

##### Material examined.

Turkey: Muğla, University campus, MT, 720 m, 37°09'42"N, 28°22'13"E, H. Kavak, 26.v.–26.vi.2015, 2M and 1F.

##### Distribution.

Holarctic species.

#### Elachiptera bimaculata

Taxon classificationAnimaliaDipteraChloropidae

(Loew, 1858)

##### Material examined.

Turkey: Toparlar, lowland forest, 36°58'39"N, 28°39'30"E, sweeping, 5.–7.5.2013, 2M; Turkey: Akyaka, pasture, 37°03'19"N, 28°20'07"E, 28.4.–8.5.2013, 6 m, 3M; Samsun, University campus, 22.vi–4.vii.2014, 1M.

##### Distribution.

southern Europe, Canary Islands, Madeira, Israel.

#### * Elachiptera brevipennis

Taxon classificationAnimaliaDipteraChloropidae

(Meigen, 1830)

##### Material examined.

Samsun, University campus, 22.vi–4.vii.2014, 1M.

##### Distribution.

widely distributed in the West Palaearctic Region.

#### Elachiptera cornuta

Taxon classificationAnimaliaDipteraChloropidae

(Fallén, 1820)

##### Material examined.

Turkey: Toparlar, lowland forest, 36°58'39"N, 28°39'30"E, sweeping, 5.–7.5.2013, 1M; Samsun, University campus, 22.vi–4.vii.2014, 1M.

##### Distribution.

widely distributed in the Palaearctic Region.

*Elachiptera graeca Becker, 1910

##### Material examined.

Turkey: Akyaka, pasture, 4 m, 37°03'09"N, 28°20'17"E, 23.–27.ix.2012, 4M and 2F; Turkey: Akyaka, pasture, 37°03'19"N, 28°20'07"E, 28.4.–8.5.2013, 6 m, 8M and 4F.

##### Distribution.

Mediterranean species

#### Elachiptera rufifrons

Taxon classificationAnimaliaDipteraChloropidae

Duda, 1932

##### Material examined.

Turkey: Akyaka, pasture, 4 m, 37°03'09"N, 28°20'17"E, 23.–27.ix.2012, 2M.

##### Distribution.

southern Eurasian species, known from Spain to China.

#### * Elachiptera sarda

Taxon classificationAnimaliaDipteraChloropidae

Nartshuk, 2009

##### Material examined.

Turkey: Akyaka, pasture, 4 m, 37°03'09"N, 28°20'17"E, 23.–27.ix.2012, 4M and 3F; Turkey: 8 km S of Çine, river bank, 68 m, 37°32'34"N, 28°03'46"E, 21.ix.2012, 2M and 1F; Turkey: Akyaka, pasture, 37°03'19"N, 28°20'07"E, 28.4.–8.5.2013, 6 m, 2M and 2F; Turkey: Samsun, University campus, 22.vi–4.vii.2014, 3M.

##### Distribution.

this species was described from Italia, Sardegna and further known from the Balearic Islands.

#### * Eribolus hungaricus

Taxon classificationAnimaliaDipteraChloropidae

Becker, 1910

##### Material examined.

Turkey: Akyaka, pasture, 4 m, 37°03'09"N, 28°20'17"E, 23.–27.ix.2012, 3M and 1F; Turkey: Akyaka, pasture, 37°03'19"N, 28°20'07"E, 28.4.–8.5.2013, 6 m, 3M and 2F.

##### Distribution.

widely distributed West Palaearctic species.

#### * Gaurax fascipes

Taxon classificationAnimaliaDipteraChloropidae

Becker, 1910

##### Material examined.

Turkey: Samsun, University campus, 22.vi–4.vii.2014, 1M.

##### Distribution.

widely distributed West Palaearctic species.

#### * Gaurax niger

Taxon classificationAnimaliaDipteraChloropidae

Czerny, 1906

##### Material examined.

Samsun, University campus, 22.vi–4.vii.2014, 1M.

##### Distribution.

widely distributed West Palaearctic species.

*Hapleginella laevifrons* (Loew, 1858)

##### Material examined.

Turkey: 11km E of Muğla, pine wood + meadow, 1310m, 37°12'45"N, 28°27'42"E, 23.v.2011, 1M.

##### Distribution.

Eurasian species

#### * Incertella zuercheri

Taxon classificationAnimaliaDipteraChloropidae

(Collin, 1946)

##### Material examined.

Turkey: Akyaka, pasture, 4 m, 37°03'09"N, 28°20'17"E, 23.–27.ix.2012, 6M and 2F; Turkey: 8 km S of Çine, river bank, 68 m, 37°32'34"N, 28°03'46"E, 21.ix.2012, 2M and 2F.

##### Distribution.

widely distributed Palaearctic species.

#### Lasiambia albidipennis

Taxon classificationAnimaliaDipteraChloropidae

(Strobl, 1893)

##### Material examined.

Turkey: Muğla, University campus, YPWT, 720 m, 37°09'42"N, 28°22'13"E, 26.–27.vi.2015, 1M; Turkey: 4 km N of Yatagan, *Foeniculus* flowers, 460 m, 37°22'12"N, 28°09'22"E, 30.vi.2015, 2F; Turkey: Akyaka, salty meadow, 2 m, 37°01'49"N, 28°20'01"E, 22.vi.–1.vii.2015, 1M.

##### Distribution.

this species is known from southern Europe, Kazakhstan, and Asia Minor.

#### Lasiambia brevibucca

Taxon classificationAnimaliaDipteraChloropidae

(Duda, 1933)

##### Material examined.

Turkey: Akyaka, pasture, 4 m, 37°03'09"N, 28°20'17"E, 23.–27.ix.2012, 1M.

##### Distribution.

this species is known from Europe, Turkey and Iran.

#### * Lasiambia coxalis

Taxon classificationAnimaliaDipteraChloropidae

(von Roser, 1840)

##### Material examined.

Turkey: Muğla, University campus, YPWT, 720 m, 37°09'42"N, 28°22'13"E, 26.–27.vi.2015, 2M.

##### Distribution.

widely distributed Palaearctic species.

#### * Lasiambia fycoperda

Taxon classificationAnimaliaDipteraChloropidae

(Becker, 1910)

##### Material examined.

Turkey: Muğla, University campus, 700 m, 37°09'41"N, 28°22'21"E, Malaise trap, edge of pine wood, xi.2012–iii.2013, 4M and 2F.

##### Distribution.

this species is known from Southern Europe

#### Lasiochaeta pubescens

Taxon classificationAnimaliaDipteraChloropidae

(Thalhammer, 1898)

##### Material examined.

Turkey: Akyaka, pasture, 4 m, 37°03'09"N, 28°20'17"E, 23.–27.ix.2012, 15M and 12F; Turkey: Akyaka, river bank + salty meadow, 37°03'16"N, 28°19'57"E, 16.–27.v.2011, 10M and 5F; Turkey: Akyaka, pasture, 37°03'19"N, 28°20'07"E, 28.4.–8.5.2013, 6 m, 4M and 2F; Samsun, University campus, 22.vi–4.vii.2014, 12M and 24F.

##### Distribution.

common and widely distributed species in the southern Palaearctic Region, from Azores and Madeira to Afghanistan, recently spreading as north as England and Northern Germany.

#### * Lipara rufitarsis

Taxon classificationAnimaliaDipteraChloropidae

Loew, 1858

##### Material examined.

Turkey: Akyaka, salty meadow, SW + PT, 37°02'53"N, 28°19'39"E, , 28.4.–9.5.2013, 1M Turkey: Toparlar, lowland forest, 36°58'39"N, 28°39'30"E, sweeping, 5.–7.5.2013, 2M.

##### Distribution.

widely distributed Holarctic species.

#### * Lipara similis

Taxon classificationAnimaliaDipteraChloropidae

Schiner, 1854

##### Material examined.

Turkey: Toparlar, lowland forest, 36°58'39"N, 28°39'30"E, sweeping, 5.–7.5.2013, 2M.

##### Distribution.

widely distributed Palaearctic species.

#### Oscinimorpha arcuata

Taxon classificationAnimaliaDipteraChloropidae

(Duda, 1932)

##### Material examined.

Turkey: Akyaka, 40 m, forest, SW + PT, 37°03'21"N, 28°19'09"E, 16.–27.v.2011, 2M and 6F.

##### Distribution.

West Palaearctic species.

#### Oscinimorpha longirostris

Taxon classificationAnimaliaDipteraChloropidae

(Loew, 1858)

##### Material examined.

Turkey: Akyaka, pasture, 37°03'19"N, 28°20'07"E, 28.4.–8.5.2013, 6 m, 2M; Turkey: Akyaka, river bank + salty meadow, 37°03'16"N, 28°19'57"E, 16.–27.v.2011, 12M and 10F.

##### Distribution.

mediterranean species, known from the Canary Islands, southern Europe, and North Africa to Israel.

#### * Oscinimorpha minutissima

Taxon classificationAnimaliaDipteraChloropidae

(Strobl, 1900)

##### Material examined.

Turkey: Akyaka, pasture, 37°03'19"N, 28°20'07"E, 28.4.–8.5.2013, 6 m. 3M; Turkey: Muğla, 700 m, University campus, SW + PT, 37°09'42"N, 28°22'21"E, 29.iv.–10.v.2013, 2M.

##### Distribution.

this species is known from North Africa and West Palaearctic Region.

#### Oscinimorpha novakii

Taxon classificationAnimaliaDipteraChloropidae

(Strobl, 1893)

##### Material examined.

Samsun, University campus, 22.vi–4.vii.2014, 1M; Turkey: Akyaka, river bank + salty meadow, 37°03'16"N, 28°19'57"E, 16.–27.v.2011, 10M and 6F.

##### Distribution.

mediterranean species, known from the Canary Islands, southern Europe to Israel.

#### Polyodaspis splendida

Taxon classificationAnimaliaDipteraChloropidae

Nartshuk, 2012

##### Material examined.

Turkey: 8 km S of Çine, river bank, 68 m, 37°32'34"N, 28°03'46"E, 21.ix.2012, 4M and 3F; Turkey: 4 km N of Yatagan, *Foeniculus* flowers, 460 m, 37°22'12"N, 28°09'22"E, 30.vi.2015, 1M

##### Distribution.

this species is known only from Turkey.

#### Polyodaspis sulcicollis

Taxon classificationAnimaliaDipteraChloropidae

(Meigen, 1838)

##### Material examined.

Turkey: 11km E of Muğla, pine wood + meadow, 1310m, 37°12'45"N, 28°27'42"E, 1.v.2013, 3M and 1F; Turkey: Samsun, University campus, 22.vi–4.vii.2014, 5M and 6F; Turkey: 4 km N of Yatagan, *Foeniculus* flowers, 460 m, 37°22'12"N, 28°09'22"E, 30.vi.2015, 3M and 4F; Turkey: 8 km S of Çine, river bank, 68 m, SW + YPWT, 37°32'34"N, 28°03'46"E, 28.–30.vi.2015, 5M and 6F

##### Distribution.

this species is distributed in Europe, the mediterranean subregion, and in Palaearctic Asia eastwards to Yakutia and Mongolia.

#### Sabroskyina aharonii

Taxon classificationAnimaliaDipteraChloropidae

(Duda, 1933)

##### Material examined.

Turkey: Akyaka, salty meadow, 2 m, 37°03'N, 28°20'E, 23.–27.ix.2012, 1M; Turkey: 8 km S of Çine, river bank, 68 m, 37°32'34"N, 28°03'46"E, 21.ix.2012, 5M; Turkey: Toparlar, lowland forest, 36°58'39"N, 28°39'30"E, sweeping, 5.–7.5.2013, 6M and 5F; Turkey: Dalyan, orchard, 4 m, 36°49'37"N, 28°39'39"E, 24.ix.2012, 32M and 43F;

##### Distribution.

the species was previously known from Turkey to Pakistan and Israel, Africa from Egypt to Chad, Seychelles, and Cape Verde Islands.

#### * Speccafrons genavensis

Taxon classificationAnimaliaDipteraChloropidae

Merz, 2008

##### Material examined.

Turkey: Akyaka, river bank + salty meadow, 37°03'16"N, 28°19'57"E, 16.–27.v.2011, 3M and 2F; Turkey: Akyaka, pasture, 4 m, 37°03'09"N, 28°20'17"E, 23.–27.ix.2012, 4M and 2 F; Turkey: 8 km S of Çine, river bank, 68 m, 37°32'34"N, 28°03'46"E, 21.ix.2012, 2M and 2F.

##### Distribution.

described and hitherto known only from Switzerland.

#### * Trachysiphonella carinifacies

Taxon classificationAnimaliaDipteraChloropidae

Nartshuk, 1964

##### Material examined.

Turkey: Akyaka, river bank + salty meadow, 37°03'16"N, 28°19'57"E, 16.–27.v.2011, 3M and 2F; Turkey: Akyaka, pasture, 4 m, 37°03'09"N, 28°20'17"E, 23.–27.ix.2012, 4M and 2F.

##### Distribution.

the species was described from Kazakhstan and further recorded from Mongolia, Tajikistan, Saudi Arabia, Yemen and Greece.

#### * Trachysiphonella recurva

Taxon classificationAnimaliaDipteraChloropidae

Deeming & Al–Dhafer, 2012

##### Material examined.

Turkey: 13km NE of Muğla, pasture/pine wood, 1200m, 37°14'50"N, 28°30'E, 23.–27.vi.2015, 1M; Turkey: 8 km S of Çine, river bank, 68 m, SW + YPWT, 37°32'34"N, 28°03'46"E, 28.–30.vi.2015, 1M and 1F.

##### Distribution.

this species was described from Yemen and further recorded from Oman and Saudi Arabia.

#### * Trachysiphonella ruficeps

Taxon classificationAnimaliaDipteraChloropidae

(Macquart, 1835)

##### Material examined.

Turkey: 8 km S of Çine, river bank, 68 m, 37°32'34"N, 28°03'46"E, 10.–12.ix.2014, 3M and 2F; Turkey: Toparlar, lowland forest, 36°58'39"N, 28°39'30"E, sweeping, 5.–7.5.2013, 2M and 1F; Turkey: Akyaka, pasture, 37°03'19"N, 28°20'07"E, 28.4.–8.5.2013, 6 m, 4M and 2F

##### Distribution.

this species is distributed in Palaearctic Region.

#### * Tricimba albiseta

Taxon classificationAnimaliaDipteraChloropidae

Dely–Draskovits, 1983

##### Material examined.

Turkey: Akyaka, river bank + salty meadow, 37°03'16"N, 28°19'57"E, 16.–27.v.2011, 1M; Turkey: Akyaka, pasture, 37°03'19"N, 28°20'07"E, 28.4.–8.5.2013, 6 m, 3M.

##### Distribution.

this species is known from Europe.

#### Tricimba humeralis

Taxon classificationAnimaliaDipteraChloropidae

(Loew, 1858)

##### Material examined.

Turkey: Muğla, University campus, YPWT, 720 m, 37°09'42"N, 28°22'13"E, 26.–27.vi.2015, 2M and 11F

##### Distribution.

widely distributed species, recorded from the southern Palaearctic Region and the Afrotropical Region.

#### * Tricimba hungarica

Taxon classificationAnimaliaDipteraChloropidae

Dely–Draskovits, 1983

##### Material examined.

Turkey: Muğla, University, campus, PT, 700 m, 37°09'42"N, 28°22'21"E, 21.–24.ix.2012, 1M.

##### Distribution.

this species is known only from Hungary, Czech Republic and Ukraine.

#### * Tricimba lineella

Taxon classificationAnimaliaDipteraChloropidae

(Fallén, 1820)

##### Material examined.

Turkey: 8 km S of Çine, river bank, 68 m, 37°32'34"N, 28°03'46"E, 21.ix.2012, 1M; Turkey: Samsun, University campus, 22.vi–4.vii.2014, 1M.

##### Distribution.

widely distributed Palaearctic species.

### Subfamily: Chloropinae

#### Assuania thalhammeri

Taxon classificationAnimaliaDipteraChloropidae

(Strobl, 1893)

##### Material examined.

Turkey: Toparlar, lowland forest, 8 m, 36°59'27"N, 28°38'50"E, 24.ix.2012, 2M; Turkey: 8 km S of Çine, river bank, 68 m, 37°32'34"N, 28°03'46"E, 10.–12.ix.2014, 4M; Turkey: 8 km S of Çine, river bank, 68 m, 37°32'34"N, 28°03'46"E, 21.ix.2012, 4M and 3F.

##### Distribution.

south Palaearctic species, known from southern Europe and North Africa to Afghanistan.

#### Camarota curvipennis

Taxon classificationAnimaliaDipteraChloropidae

(Latreille, 1805)

##### Material examined.

Samsun, University campus, 22.vi–4.vii.2014, 2M.

##### Distribution.

This species is known almost from all Europe (except the northern parts), the Caucasus, southern part of Palaearctic Asia and North Africa.

#### Cetema neglectum

Taxon classificationAnimaliaDipteraChloropidae

Tonnoir, 1921

##### Material examined.

Turkey: Akyaka, pasture, 37°03'19"N, 28°20'07"E, 28.4.–8.5.2013, 6 m, 2M; Samsun, University campus, 22.vi–4.vii.2014, 1M.

##### Distribution.

this species is known only from Europe and Turkey.

#### * Chlorops figuratus

Taxon classificationAnimaliaDipteraChloropidae

(Zetterstedt, 1848)

##### Material examined.

Samsun, University campus, 22.vi–4.vii.2014, 1M.

##### Distribution.

widely distributed Palaearctic species.

#### Chlorops freidmani

Taxon classificationAnimaliaDipteraChloropidae

Nartshuk, 2012

##### Material examined.

Turkey: Akyaka, pasture, 37°03'19"N, 28°20'07"E, 28.4.–8.5.2013, 6 m, 4M and 2F; Turkey: Toparlar, lowland forest, 36°58'39"N, 28°39'30"E, sweeping, 5.–7.5.2013, 5M and 3F.

##### Distribution.

this species is known only from Turkey.

#### * Chlorops geminatus

Taxon classificationAnimaliaDipteraChloropidae

Meigen, 1830

##### Material examined.

Samsun, University campus, 22.vi–4.vii.2014, 1M.

##### Distribution.

this species is distributed in Palaearctic Region.

#### * Chlorops hypostigma

Taxon classificationAnimaliaDipteraChloropidae

Meigen, 1830

##### Material examined.

Samsun, University campus, 22.vi–4.vii.2014, 1M; Turkey: Toparlar, lowland forest, SW + YPWT, 8 m, 36°59'27"N, 28°38'50"E, 22.–24.vi.2015, 2M and 1F;

##### Distribution.

Palaearctic Region.

#### * Chlorops interruptus

Taxon classificationAnimaliaDipteraChloropidae

Meigen, 1830

##### Material examined.

Samsun, University campus, 22.vi–4.vii.2014, 3M.

##### Distribution.

this species is known from Palaearctic Region.

#### * Chlorops limbatus

Taxon classificationAnimaliaDipteraChloropidae

Meigen, 1830

##### Material examined.

Turkey: Akyaka, pasture, 37°03'19"N, 28°20'07"E, 28.4.–8.5.2013, 6 m, 2M; Turkey: Akyaka, river bank + salty meadow, 37°03'16"N, 28°19'57"E, 16.–27.v.2011, 8M and 6F.

##### Distribution.

widely distributed Palaearctic species.

#### Chlorops pumilionis

Taxon classificationAnimaliaDipteraChloropidae

(Bjerkander, 1778)

##### Material examined.

Turkey: Muğla, 700 m, University campus, SW + PT, 37°09'42"N, 28°22'21"E, 29.iv.–10.v.2013, 1F; Turkey: 15km SW of Muğla, damp valley nr.brook, 630 m, 37°06'31"N, 28°15'31"E, 23.v.20111M.

##### Distribution.

Eurasian species, known from Europe to Mongolia.

#### * Chlorops serenus

Taxon classificationAnimaliaDipteraChloropidae

Loew 1866

##### Material examined.

Turkey: Akyaka, forest, 37°03'16"N, 28°19'35"E, 30.4.–9.5.2013, 30 m, 1M.

##### Distribution.

this species is known from West Palaearctic Region.

#### * Chloropsina lucens

Taxon classificationAnimaliaDipteraChloropidae

(Becker, 1910)

##### Material examined.

Turkey: Akyaka, river bank + salty meadow, 37°03'16"N, 28°19'57"E, 16.–27.v.2011, 1F; Turkey: Akyaka, pasture, 4 m, 37°03'09"N, 28°20'17"E, 23.–27.ix.2012, 1F; Turkey: Akyaka, salty meadow, SW + PT, 37°02'53"N, 28°19'39"E, 28.4.–9.5.2013, 1F; Turkey: Akyaka, pasture, 4 m, 37°03'09"N, 28°20'17"E, 8.–14.ix.2014, 1M.

##### Distribution.

this species was described and hitherto known only from Greece.

#### * Cryptonevra diadema

Taxon classificationAnimaliaDipteraChloropidae

(Meigen 1830)

##### Material examined.

Turkey: Akyaka, salty meadow, SW + PT, 37°02'53"N, 28°19'39"E, 28.4.–9.5.2013, 1M; Turkey: 8 km S of Çine, river bank, 68 m, 37°32'34"N, 28°03'46"E, 21.ix.2012, 3M.

##### Distribution.

species widely distributed in North Africa and Palaearctic Region.

#### * Cryptonevra flavitarsis

Taxon classificationAnimaliaDipteraChloropidae

(Meigen, 1830)

##### Material examined.

Turkey: Akyaka, salty meadow, SW + PT, 37°02'53"N, 28°19'39"E, 28.4.–9.5.2013, 2M and 1F; Turkey: Akyaka, pasture, 37°03'19"N, 28°20'07"E, 28.4.–8.5.2013, 6 m, 3M and 2F; Turkey: Akyaka, river bank + salty meadow, 37°03'16"N, 28°19'57"E, 16.–27.v.2011, 4M; Turkey: Toparlar, lowland forest, SW + YPWT, 8 m, 36°59'27"N, 28°38'50"E, 22.–24.vi.2015, 4M and 2F.

##### Distribution.

Europe and Kazakhstan.

#### Cryptonevra nigritarsis

Taxon classificationAnimaliaDipteraChloropidae

(Duda, 1933)

##### Material examined.

Turkey: Akyaka, river bank + salty meadow, 37°03'16"N, 28°19'57"E, 16.–27.v.2011, 1M.

##### Distribution.

Palaearctic distributed species.

#### Diplotoxa messoria

Taxon classificationAnimaliaDipteraChloropidae

(Fallén, 1820)

##### Material examined.

Turkey: Akyaka, pasture, 37°03'19"N, 28°20'07"E, 28.4.–8.5.2013, 6 m, 1M.

##### Distribution.

Holarctic species; in the Palaearctic Region known from the British Isles to Far East Russia.

#### Eurina ducalis

Taxon classificationAnimaliaDipteraChloropidae

A. Costa, 1885

##### Material examined.

Turkey: Toparlar, lowland forest, 36°58'39"N, 28°39'30"E, sweeping, 5.–7.5.2013, 4M and 4F; Turkey: Akyaka, pasture, 37°03'19"N, 28°20'07"E, 28.4.–8.5.2013, 6 m, 4M and 4F.

##### Distribution.

this species is known from Central and South Europe, Syria and Israel.

#### * Eurina lurida

Taxon classificationAnimaliaDipteraChloropidae

Meigen, 1830

##### Material examined.

Turkey: Akyaka, pasture, 37°03'19"N, 28°20'07"E, 28.4.–8.5.2013, 6 m, 1M.

##### Distribution.

widely distributed Palaearctic species known also from Near East.

#### Eurina triangularis

Taxon classificationAnimaliaDipteraChloropidae

Becker, 1903

##### Material examined.

Turkey: Akyaka, pasture, 37°03'19"N, 28°20'07"E, 28.4.–8.5.2013, 6 m, 1M.

##### Distribution.

this species is known from North Africa (Egypt) and Israel.

#### * Eutropha fulvifrons

Taxon classificationAnimaliaDipteraChloropidae

(Haliday, 1833)

##### Material examined.

Turkey: Akyaka, salty meadow, SW + PT, 37°02'53"N, 28°19'39"E, 28.4.–9.5.2013, 3M

##### Distribution.

the species is known in Near East and West Palaearctic Region.

#### * Lagaroceras megalops

Taxon classificationAnimaliaDipteraChloropidae

Becker, 1903

##### Material examined.

Turkey: 8 km S of Çine, river bank, 68 m, 37°32'34"N, 28°03'46"E, 10.–12.ix.2014, 2M

##### Distribution.

this species is known from Near East (Egypt), Ethiopia, Mozambique and South Africa.

#### * Lasiosina albipila

Taxon classificationAnimaliaDipteraChloropidae

(Loew, 1866)

##### Material examined.

Turkey: Akyaka, pasture, 4 m, 37°03'09"N, 28°20'17"E, 23.–27.ix.2012, 3M and 1F; Turkey: Akyaka, river bank + salty meadow, 37°03'16"N, 28°19'57"E, 16.–27.v.2011, 2M and 1F.

##### Distribution.

Palaearctic species.

#### * Lasiosina aurea

Taxon classificationAnimaliaDipteraChloropidae

Dely-Draskovits, 1981

##### Material examined.

Turkey: 8 km S of Çine, river bank, 68 m, 37°32'34"N, 28°03'46"E, 21.ix.2012, 1M; Turkey: Akyaka, pasture, 37°03'19"N, 28°20'07"E, 28.4.–8.5.2013, 6 m, 2M; Turkey: Toparlar, lowland forest, 36°58'39"N, 28°39'30"E, sweeping, 5.–7.5.2013; Turkey: Toparlar, lowland forest, 8 m, 36°59'27"N, 28°38'50"E, 24.ix.2012, 3M and 3F.

##### Distribution.

this species was described from Israel.

#### * Lasiosina cinctipes

Taxon classificationAnimaliaDipteraChloropidae

(Meigen, 1830)

##### Material examined.

Turkey: Samsun, University campus, 22.vi–4.vii.2014, 4M.

##### Distribution.

Palaearctic species.

#### Lasiosina emiliae

Taxon classificationAnimaliaDipteraChloropidae

Dely-Draskovits, 1982

##### Material examined.

Turkey: Akyaka, salty meadow, SW + PT, 37°02'53"N, 28°19'39"E, 28.4.–9.5.2013, 2M.

##### Distribution.

this species was known earlier from Kazakhstan, Kirghizia, Tajikistan, and Uzbekistan.

#### Lasiosina herpini

Taxon classificationAnimaliaDipteraChloropidae

(Guérin-Méneville, 1843)

##### Material examined.

Turkey: Samsun, University campus, 22.vi–4.vii.2014, 1M.

##### Distribution.

Transpalaearctic species.

#### * Lasiosina immaculata

Taxon classificationAnimaliaDipteraChloropidae

Becker, 1912

##### Material examined.

Turkey: Akyaka, pasture, 4 m, 37°03'09"N, 28°20'17"E, 23.–27.ix.2012, 4M.

##### Distribution.

this species was known earlier from Europe and Near East.

#### * Lasiosina lindbergi

Taxon classificationAnimaliaDipteraChloropidae

(Duda, 1933)

##### Material examined.

Turkey: Toparlar, lowland forest, 36°58'39"N, 28°39'30"E, sweeping, 5.–7.5.2013, 5M.

##### Distribution.

Mediterranean species, known from Bulgaria, the North Africa and Corsica.

#### * Lasiosina paralittoralis

Taxon classificationAnimaliaDipteraChloropidae

Dely-Draskovits, 1981

##### Material examined.

Turkey: Akyaka, pasture, 4 m, 37°03'09"N, 28°20'17"E, 8.–14.ix.2014, 8M and 4F; Turkey: Toparlar, lowland forest, 8 m, 36°59'27"N, 28°38'50"E, 24.ix.2012, 6M and 8F.

##### Distribution.

this species was described from Israel.

#### * Meromyza eduardi

Taxon classificationAnimaliaDipteraChloropidae

Hubicka, 1966

##### Material examined.

Turkey: Akyaka, salty meadow, 2 m, 37°01'49"N, 28°20'01"E, 22.vi.–1.vii.2015, 2M and 1F.

##### Distribution.

this species was known earlier from Estonia, Lithuania and Poland.

#### Meromyza filippovi

Taxon classificationAnimaliaDipteraChloropidae

Ozerov, 2009

##### Material examined.

Turkey: Toparlar, lowland forest, 8 m, 36°59'27"N, 28°38'50"E, 24.ix.2012, 4M and 1F.

##### Distribution.

this species is known only from European part of Turkey.

#### * Meromyza meigeni

Taxon classificationAnimaliaDipteraChloropidae

Nartshuk, 2006

##### Material examined.

Turkey: 13km NE of Muğla, pasture/pine wood, 1200m, 37°14'50"N, 28°30'E, 23.–27.vi.2015, 6M and 9F.

##### Distribution.

this species was described from Slovenia and further known from Bulgaria, Albania, Macedonia and Bosnia.

#### * Meromyza pluriseta

Taxon classificationAnimaliaDipteraChloropidae

Peterfi, 1961

##### Material examined.

Turkey: Gökçeova Gölü, lake shore, 1 750 m, 37°03'42.52"N, 28°48'28.42"E, 20.ix.2012, 6M; Turkey: 8 km S of Çine, river bank, 68 m, 37°32'34"N, 28°03'46"E, 21.ix.2012, 4M and 2F.

##### Distribution.

Palaearctic species.

#### Meromyza nigriventris

Taxon classificationAnimaliaDipteraChloropidae

Macquart, 1835

##### Material examined.

Samsun, University campus, 22.vi–4.vii.2014, 1M.

##### Distribution.

Holarctic species: in the Palaearctic Region it is widely distributed from the British Isles to Japan; in North America it is known only from the West.

#### Phyladelphus thalhammeri

Taxon classificationAnimaliaDipteraChloropidae

Becker, 1910

##### Material examined.

Turkey: Akyaka, pasture, 4 m, 37°03'09"N, 28°20'17"E, 8.–14.ix.2014, 6M and 4F; Turkey: 8 km S of Çine, river bank, 68 m, 37°32'34"N, 28°03'46"E, 10.–12.ix.2014, 5M and 4F;

##### Distribution.

Mediterranean species.

#### Thaumatomyia notata

Taxon classificationAnimaliaDipteraChloropidae

(Meigen, 1830)

##### Material examined.

Turkey: Akyaka, forest, 37°03'16"N, 28°19'35"E, 30.4.–9.5.2013, 30 m, 4M and 8F; Turkey: Toparlar, lowland forest, 36°58'39"N, 28°39'30"E, sweeping, 5.–7.5.2013, 2M and 8F; Turkey: Akyaka, pasture, 37°03'19"N, 28°20'07"E, 28.4.–8.5.2013, 6 m, 1M and 6F; Samsun, University campus, 22.vi–4.vii.2014, 4M and 8F.

##### Distribution.

widespread species, recorded from the Palaearctic, Afrotropical, and Oriental Regions.

#### Thaumatomyia sulcifrons

Taxon classificationAnimaliaDipteraChloropidae

(Becker, 1907)

##### Material examined.

Turkey: 4 km N of Yatagan, *Foeniculus* flowers, 460 m, 37°22'12"N, 28°09'22"E, 30.vi.2015, 1M and 2F.

##### Distribution.

South Palaearctic species, known from the Canary Islands to China.

### Descriptions of new species

#### Oscinellinae

##### Dicraeus civeleki sp. n.

Taxon classificationAnimaliaDipteraChloropidae

http://zoobank.org/DB8D5B04-4123-4738-9552-5D377D1C4D75

Figs 1–3 , 4–5

###### Type material.

Holotype male, Turkey: Akyaka, salty meadow, 2 m, 37°01'49"N, 28°20'01"E, 22.vi.–1.vii.2015. Holotype is in good condition, abdomen on plastic tags together with the specimen. Paratype: 1M same data.

**Figure 1–3.**
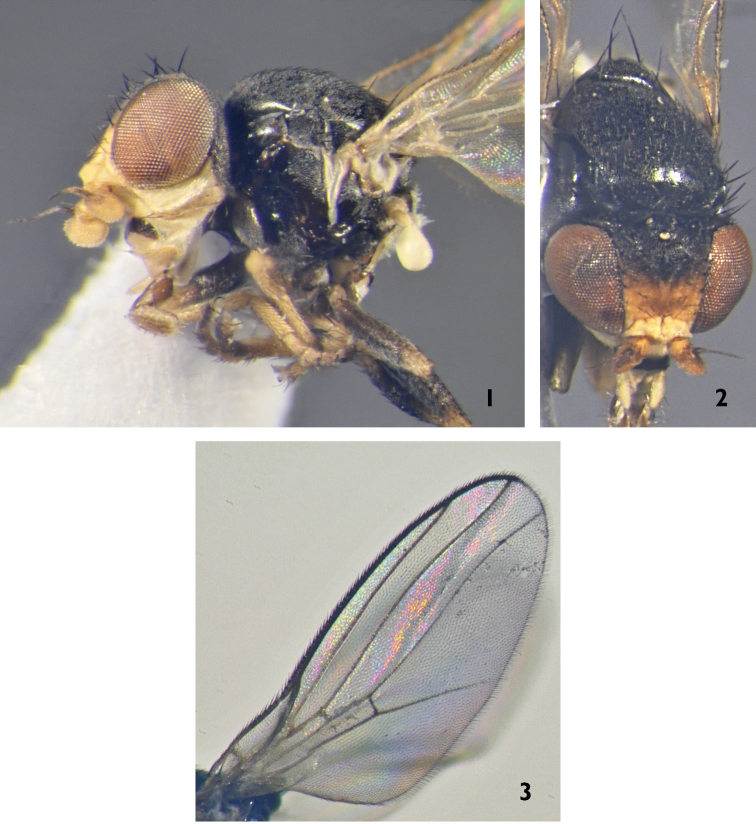
*Dicraeus civeleki* sp. n. (holotype): **1** body (abdomen missing), lateral view **2** body (abdomen missing), dorsal view **3** wing.

###### Diagnosis.

Grey dusted black species with yellow face, anterior part of frons, antennae, palpus, fore and mid tibia. Costal vein reaches one-fourth the way between R_4 + 5_ and M_1 + 2_.

**Figure 4–9.**
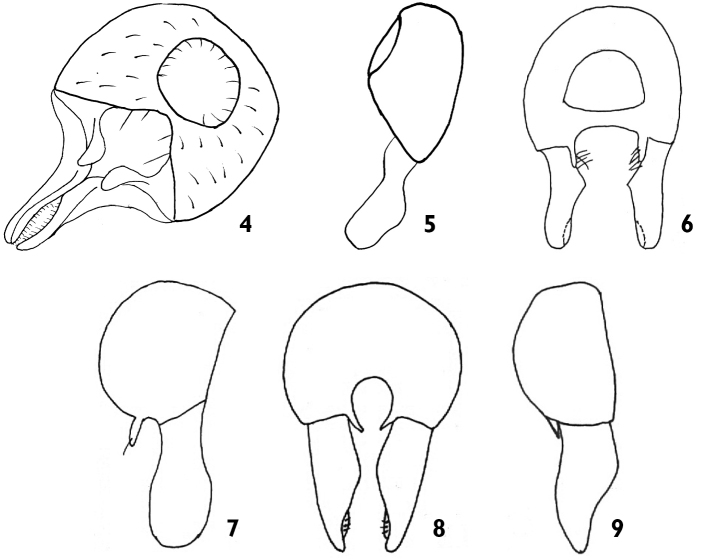
**4–5** *Dicraeus civeleki* sp. n. (holotype): **4** epandrium posterior view **5** epandrium lateral view **6–7** *Dicraeus beschovski*: **6** epandrium posterior view **7** epandrium lateral view (after Nartshuk 2010) **8–9** *Dicraeus sabroskyi*: **8** epandrium posterior view **9** epandrium lateral view (after Nartshuk 2010).

###### Description.

*Male*. Frons longer than wide, yellow on anterior third and black on posterior portion, ocellar triangle black, 2/3 length of frons. Face and gena yellow. Gena wider than first flagellomere with a row of black peristomal setulae. Palpus yellow with black setulae. Antenna yellow, first flagellomere round and yellow, arista short pubescent. Occiput black. Setae and setulae of head black.

*Thorax* black with grey microtrichosity, entirely covered with black setulae. Scutellum round triangular with long apical convergent setae and a pair of subapicals 2/3 length of apical ones. Anterior portion of pleura shining, anepisternum and katepisternum partly microtrichose. Chaetotaxy: 2 postpronotal, 1 + 2 notopleural, two postalar and one prescutellar setae. Wing clear with whitish yellow veins. Costal vein reaches one-fourth the length between R_4 + 5_ and M_1 + 2_ (Fig. 3). Halter whitish yellow. Legs: fore coxa, fore and mid tibia yellow, all femora and hind tibia black.*Abdomen* brown with a narrow yellow band on tergites. Male genitalia (Figs 4–5): epandrium black, surstylus brownish yellow with several long setae at base. Apex of surstylus broad and straight. Cercus broad and orthogonally curved, not pointed.

*Body length*: 2 mm.

*Female*: unknown.

###### Remarks.

The species belongs to subgenus Oedesiella Becker based on the structure of the male genitalia: cerci long and wide apart, surstyli longer than epandrium. Cerci wide and curved, not narrow, straight and pointed, surstylus with wide and straight apex, not narrowed as in *D. sabroskyi* Beschovski, 1977 (Figs 8–9) and not rounded as in *D. beschovski* Nartshuk, 2010 (Figs 6–7).

###### Etymology.

Named in honour of Prof. Hasan Civelek, our colleague and dipterologist from Muğla University, Turkey.

### Chloropinae

#### Meromyza samsunensis sp. n.

Taxon classificationAnimaliaDipteraChloropidae

http://zoobank.org/F6774AC1-8773-4C3B-9927-A02419668DCA

Figs 10–12 , 13–15

##### Type material.

Holotype male, Turkey: Samsun, University campus, 22.vi–4.vii.2014. Holotype is in good condition, abdomen on plastic tags together with the specimen. Paratypes: 2M and 2F same data.

**Figure 10–12.**
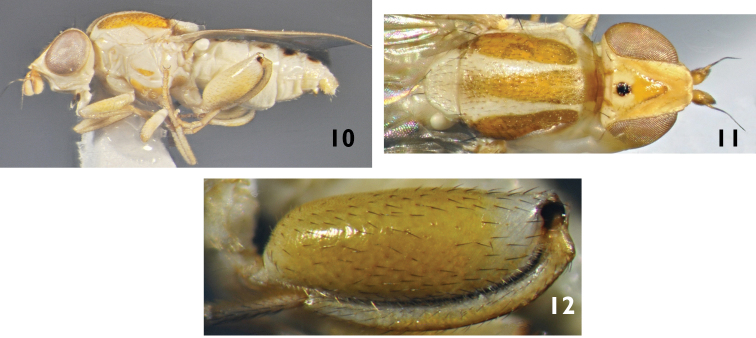
*Meromyza samsunensis* sp. n. (paratype): **10** body lateral view **11** body dorsal view **12** hind femora, lateral (dorsal) view.

##### Diagnosis.

Species with black palpus on apical half, first flagellomere 1.5 times as long as wide, red grey microtrichose stripes on scutum and hind femur nearly four times thicker than tibia. *Meromyza samsunensis* has anterior process of postgonite widened laterally forming distinct longitudinal rib; upper half parallel and curved, lower half concave. This character is hardly visible in lateral view (Fig. 15). In *M. femorata*, the anterior process of postgonite is flat, wide and with three to four smooth spinules on the surface (Fig. 16).

**Figure 13–16.**
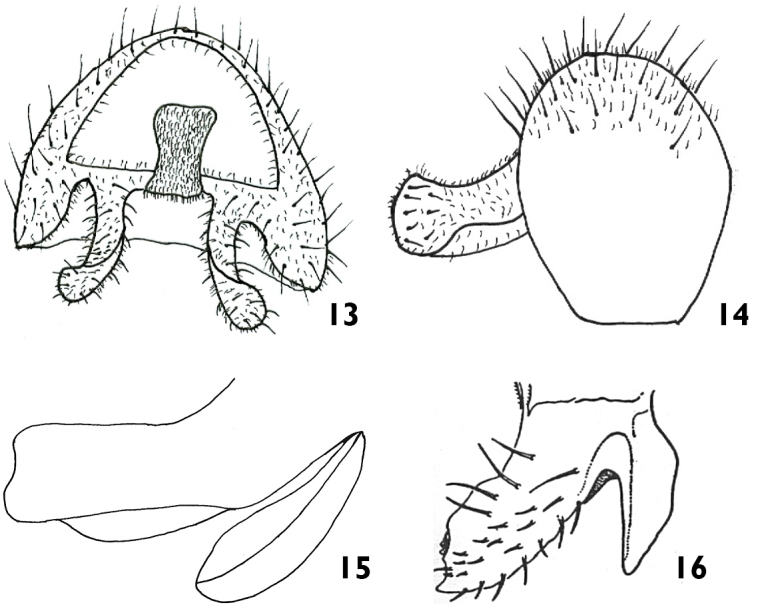
*Meromyza samsunensis* sp. n. (holotype): **13** epandrium, posterior view **14** epandrium, lateral view **15** postgonite, lateral view **16** *Meromyza femorata*: postgonite, lateral view (after Nartshuk and Fedoseeva, 2011).

##### Description.

*Male* (Figs 10–11). Ground colour yellow. Frons produced anteriorly, produced region of frons same width of first flagellomere. Ocellar triangle occupying two-thirds of frons, shining, rugose on apical portion and black on ocellar tubercle only, with one row of black interfrontal setae along sides. First flagellomere 1.5 times as long as wide, yellow, darkened on dorsal portion and with long pale setulae. Arista yellow, nearly bare. Genal as wide as first flagellomere. Vibrissal angle obtuse. Palpus black on apical half and yellow basally.

*Thorax*: Scutum with red grey microtrichose stripes, midstripe reaching scutellum and scutellum with small red mark. Pleura with red marks except small black mark on anepisternum. Wing hyaline with whitish yellow veins. Halter whitish yellow. Legs yellow, fore tarsus darkened. Hind femur strongly swollen, nearly four times as thick as tibia (Fig. 12).

*Abdomen*: yellow with dark midstripe and small spots on tergites 2–5. Male genitalia (Figs 13–14): epandrium yellow, with long curved surstylus evenly covered with small setulae. The upper half of anterior process of postgonite is parallel and curved, lower half concave. Posterior process enlarged (Fig. 15).

*Body length* 3.5–4.0 mm.

##### Remarks.

New species has elongated first flagellomere. The character is rear in *Meromyza*, only two species have elongated first flagellomere: *Meromyza mirabilis* Fedoseeeva, 1974 and *Meromyza longicornis* (Frey, 1921). *Meromyza mirabilis* has first flagellomere 1.5 times as long as wide (similar to *M. samsunensis* sp. n) but palpus is yellow and stripes on the scutum are brown. *Meromyza longicornis* has first flagellomere 2.5 times as long as wide and hind femur 3 times as wide as hind tibia. *M. samsunensis* sp. n is similar to *Meromyza femorata* Macquart, 1835 in having red stripes on the scutum with median stripe reaching the scutellum, palpus black on apical half, and hind femur strongly swollen. The main difference between both species is in the shape of postgonite.

##### Etymology.

the species epithet refers to the location where the holotype was collected (the city of Samsun).

### Comments

The new species may be included in the key to Palaearctic species of the genus *Meromyza* Meigen (Nartshuk and Fedoseeva, 2011) by the following modification:

**Table:** 

123 (124)	Hind femur strongly thickened, at least 4 times as wide as hind tibia. Stripes of scutum rufous	**123a**
123a	Anterior process of postgonite flat, wide and with three to four smooth spikes on surface (Fig. 16)	***M. femorata***
123b	Anterior process of postgonite widened laterally forming distinct longitudinal rib; upper half parallel and curved, lower half concave. (Fig. 15)	***M. samsunensis* sp. n.**
124 (123)	Hind femur moderately thickened, less than 3 times as wide as hind tibia. Stripes of scutum mostly dark; if rufus, anterior margin of anterior process of postgonite sharply narrowed and projecting.

## Discussion

Altogether 114 species of the family Chloropidae are known at the present time from Turkey. Nine genera (*Calamoncosis*, *Eribolus*, *Gaurax*, *Incertella*, *Speccafrons*, *Trachysiphonella*, *Chloropsina*, *Eutropha*, and *Lagaroceras*) and 46 species are recorded here for the first time. Two species (*Dicraeus civeleki* sp. n. and *Meromyza samsunensis* sp. n.) are described. Based on comparisons with the Chloropidae fauna of some adjacent countries, it seems as though the number of Chloropidae species in Turkey is in fact much larger: Bulgaria (Beschovski 1985) with 144 species, Israel with more than 100 species (Kaplan 1977), 51 species from Greece (Nartshuk 2010) and 394 species are known to occur in Europe (Nartshuk 2013).

## Supplementary Material

XML Treatment for Apotropina longepilosa

XML Treatment for Siphonellopsis lacteibasis

XML Treatment for Rhodesiella fedtshenkoi

XML Treatment for Scoliophthalmus civeleki

XML Treatment for Scoliophthalmus trapezoides

XML Treatment for Aphanotrigonum bicolor

XML Treatment for Aphanotrigonum femorellum

XML Treatment for Aphanotrigonum inerme

XML Treatment for Aphanotrigonum parahastatum

XML Treatment for Calamoncosis duinensis

XML Treatment for Conioscinella frontella

XML Treatment for Dicraeus (Dicraeus)

XML Treatment for Dicraeus beschovskii

XML Treatment for Dicraeus raptus

XML Treatment for Dicraeus tibialis

XML Treatment for Elachiptera bimaculata

XML Treatment for Elachiptera brevipennis

XML Treatment for Elachiptera cornuta

XML Treatment for Elachiptera rufifrons

XML Treatment for Elachiptera sarda

XML Treatment for Eribolus hungaricus

XML Treatment for Gaurax fascipes

XML Treatment for Gaurax niger

XML Treatment for Incertella zuercheri

XML Treatment for Lasiambia albidipennis

XML Treatment for Lasiambia brevibucca

XML Treatment for Lasiambia coxalis

XML Treatment for Lasiambia fycoperda

XML Treatment for Lasiochaeta pubescens

XML Treatment for Lipara rufitarsis

XML Treatment for Lipara similis

XML Treatment for Oscinimorpha arcuata

XML Treatment for Oscinimorpha longirostris

XML Treatment for Oscinimorpha minutissima

XML Treatment for Oscinimorpha novakii

XML Treatment for Polyodaspis splendida

XML Treatment for Polyodaspis sulcicollis

XML Treatment for Sabroskyina aharonii

XML Treatment for Speccafrons genavensis

XML Treatment for Trachysiphonella carinifacies

XML Treatment for Trachysiphonella recurva

XML Treatment for Trachysiphonella ruficeps

XML Treatment for Tricimba albiseta

XML Treatment for Tricimba humeralis

XML Treatment for Tricimba hungarica

XML Treatment for Tricimba lineella

XML Treatment for Assuania thalhammeri

XML Treatment for Camarota curvipennis

XML Treatment for Cetema neglectum

XML Treatment for Chlorops figuratus

XML Treatment for Chlorops freidmani

XML Treatment for Chlorops geminatus

XML Treatment for Chlorops hypostigma

XML Treatment for Chlorops interruptus

XML Treatment for Chlorops limbatus

XML Treatment for Chlorops pumilionis

XML Treatment for Chlorops serenus

XML Treatment for Chloropsina lucens

XML Treatment for Cryptonevra diadema

XML Treatment for Cryptonevra flavitarsis

XML Treatment for Cryptonevra nigritarsis

XML Treatment for Diplotoxa messoria

XML Treatment for Eurina ducalis

XML Treatment for Eurina lurida

XML Treatment for Eurina triangularis

XML Treatment for Eutropha fulvifrons

XML Treatment for Lagaroceras megalops

XML Treatment for Lasiosina albipila

XML Treatment for Lasiosina aurea

XML Treatment for Lasiosina cinctipes

XML Treatment for Lasiosina emiliae

XML Treatment for Lasiosina herpini

XML Treatment for Lasiosina immaculata

XML Treatment for Lasiosina lindbergi

XML Treatment for Lasiosina paralittoralis

XML Treatment for Meromyza eduardi

XML Treatment for Meromyza filippovi

XML Treatment for Meromyza meigeni

XML Treatment for Meromyza pluriseta

XML Treatment for Meromyza nigriventris

XML Treatment for Phyladelphus thalhammeri

XML Treatment for Thaumatomyia notata

XML Treatment for Thaumatomyia sulcifrons

XML Treatment for Dicraeus civeleki

XML Treatment for Meromyza samsunensis
